# Effects of e-Health Training and Social Support Interventions for Informal Caregivers of People with Dementia—A Narrative Review

**DOI:** 10.3390/ijerph18157728

**Published:** 2021-07-21

**Authors:** Esther Sitges-Maciá, Beatriz Bonete-López, Antonio Sánchez-Cabaco, Javier Oltra-Cucarella

**Affiliations:** 1 Department of Health Psychology, Miguel Hernandez University of Elche, 03202 Elche, Spain; esther.sitges@umh.es (E.S.-M.); joltra@umh.es (J.O.-C.); 2 Psychology Department, Pontifical University of Salamanca, 37002 Salamanca, Spain; asanchezca@upsa.es

**Keywords:** e-health, informal caregivers, dementia

## Abstract

Along with the burden commonly experienced by informal caregivers (ICs) of people with dementia (PwD), associated with the progressive decline that accompanies dementia, the lockdown due to the public health crisis has had a great negative impact on the emotional wellbeing, physical health, and social relationships of ICs. Support interventions through telemedicine represent an opportunity for ICs to learn the skills required for the care and maintenance of social networks. In this work, a narrative review of the effects of e-health training and social support interventions was carried out. A literature search was conducted using the ProQuest, Ovid, and Scopus databases. Information regarding social support (SS), psychological interventions, and training for the management of medications and behavioral changes was extracted. One hundred and nine studies were included in this review. Forums and training platforms were the main tools for ICs. The most effective platforms to improve SS include the participation of both ICs and health professionals. However, no significant improvements in objective caring skills were identified. Platforms developed specifically for ICs should be based in tools that ICs are familiar with, because many ICs have not yet incorporated Information and Communication Technologies in many activities of their daily lives. Education in the digitalization to ICs of PwD should be one of the priority objectives in telehealth interventions.

## 1. Introduction

The increase in the number of people over the age of 60 is now a sociodemographic reality on a global scale, revealing a large population heterogeneous in their social, emotional, and health characteristics [1]. As a consequence of the public health crisis we are experiencing, we have seen how autonomous elderly have shown more adaptive responses than other age groups [2]. However, the lockdown and the resulting restrictions have increased the vulnerability in the social and cognitive spheres of an important group of elderly people with pathologies, besides worsening their neuropsychiatric symptoms [3]. The latter has also had an important impact on the emotional burden perceived by informal caregivers (ICs) [4], with clinical consequences such as high rates of depression [5]. Indeed, the continuous decline that accompanies dementia has an impact on the physical and mental health, the general wellbeing, and the social relationships of ICs [6,7].

In the field of telemedicine, online support platforms for ICs of PwD have been in development for more than 20 years, given the limited time available to ICs and the mobility limitations that PwD may have. These platforms represent an opportunity not only to acquire the knowledge, skills, and strategies required to understand and develop the task of caring for PwD, but also to establish and maintain social contact with health professionals and other family members or caregivers living in similar circumstances. Their effectiveness has been shown for both patients through computerized stimulation programs [8] and their caregivers in dimensions such as resilience [9].

From the different reviews undertaken on the benefits of online support intervention programs for ICs of PwD, it is concluded that multicomponent interventions are the most beneficial [10,11,12,13,14]. These have a maximum duration of 9 weeks and aim at improving ICs’ quality of life, including their coping strategies and self-efficacy with regard to care [15], by increasing knowledge about dementia care and promote social support (SS) for ICs [16]. Most ICs are older women, with an average age of around 60 years [17,18,19], who require some training with this type of platform [19] to reduce the barriers caused by the Information and Communication Technologies (ICT) [20]. Likewise, it is necessary to improve aspects such as monitoring the effects of interventions in the long term [21], including a control group [13], ensuring that more samples are used, and homogenizing the interventions in terms of methodologies used to facilitate their comparison [22,23].

This SARS-CoV-2 pandemic, and the lockdown associated with it in several countries (e.g., Spain), besides having a negative impact on the psychological well-being of ICs [24,25,26], has exposed the shortcomings that exist in healthcare structures such as Alzheimer associations and day care and residential centers, revealing that these are insufficient for tackling a situation that requires providing greater protection to the most vulnerable elderly [27]. In line with the third goal of the UN’s Agenda 2030 for Sustainable Development ”Ensure healthy lives and promote wellbeing for all at all ages” [28], we need to develop strategies to adapt and strengthen social and healthcare for the vulnerable elderly population such as people with dementia (PwD) and their ICs. These reasons, now more than ever, justify the use of Online Support Interventions for Family Caregivers of PwD [29]. For this reason, we conducted an updated narrative review of the effects that e-health training and SS interventions have on ICs of PwD.

## 2. Materials and Methods

A literature search was conducted in the ProQuest, Ovid, and Scopus databases using a combination of the terms “informal”, “caregiver”, “telemedicine”, “internet”, “online”, “e-health”, “intervention”, “support”, “training”, and “dementia”. As an example, the ProQuest search “ab,ti(informal AND (caregiver OR carer* OR caregiving) AND (internet OR telemed* OR online OR on-line OR e-health OR electr*) AND (intervention OR support OR training) AND dementia)” gave 59 results. Articles were included if they (a) focused on ICs of PwD and (b) used telemedicine programs. Only articles published in peer-reviewed journals were considered in this review. Potential articles were excluded if (a) they were written in languages other than English or Spanish or (b) did not report data for review. The references of the included articles were searched for additional potential articles.

The abstract of 10 randomly selected potential articles was screened simultaneously by three authors (E.S.M., B.B.L., J.O.C.), and the Cohen’s Kappa coefficient for inter-rater reliability was calculated using the SPSS v.26 statistical package. The inter-rater reliability is the consistency of the ratings among different assessors. The Cohen’s Kappa coefficient measures whether the degree of consistency among raters is higher than what would be expected by chance and is interpreted as slight, fair, moderate, substantial, and almost perfect agreement when values are 0–0.20, 0.21–0.40, 0.41–0.60, 0.61–0.80, and 0.80–1, respectively [30]. Duplicate identification and removal, as well as application of inclusion and exclusion criteria, were performed using CADIMA [31], an open access online tool designed to assist throughout the review process.

Included articles after the abstract screening stage were rated by the three researchers, and information regarding SS, psychological interventions, and training for medication management and behavioral changes was extracted. Inconsistencies were resolved by discussion among the researchers. Articles were excluded if no data were available to interpret the effects of the online intervention for ICs.

## 3. Results

**Figure 1:**
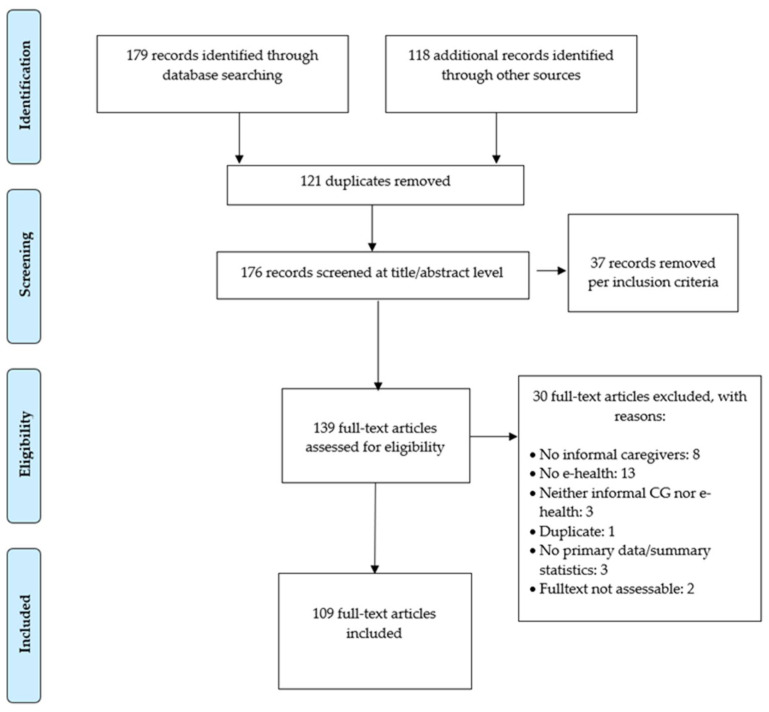
Flow chart.

The literature search gave 179 potential articles plus 118 additional records. Among these, 121 were duplicates. Of the 176 potential candidates, 109 studies were eventually included in this review (Figure 1). Cohen’s Kappa coefficient for the title and the full-text screening was 0.72 (*p* = 0.01) and 0.81 (*p* = 0.001), respectively, indicating a substantial agreement.

The results chapter is divided into two sections: the first Section 3.1 provides results regarding tools used for training ICs in caregiving situations, whereas the second Section 3.2 provides results regarding online interventions focused on improving SS for ICs of PwD. In order to ease the readiness of this chapter, the information related to each of the training programs is reported following the same structure: design, results and conclusions.

**Figure 2:**
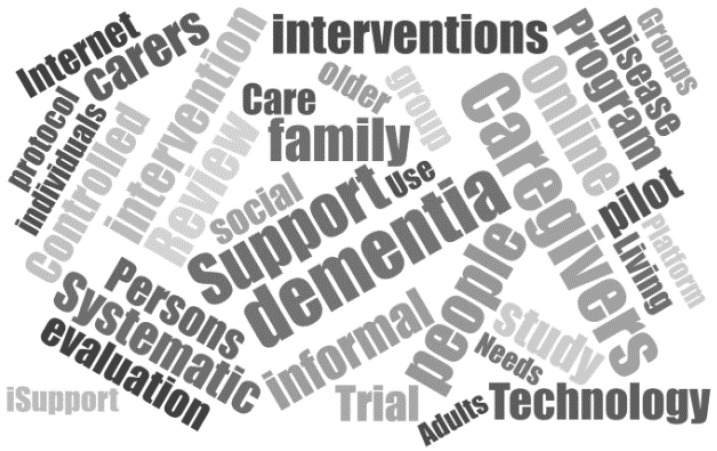
Word cloud.

In addition, a word cloud was created to graphically display the most popular terms obtained in this search, with the titles of the 139 articles initially chosen (Figure 2). This was performed by downloading the references from CADIMA and entering the titles in the online tool http://jasondavies.com (accessed on 15 July 2021). For the word cloud to be clarifying, we used the 50 most repeated words, among which are SS.

**Figure 3:**
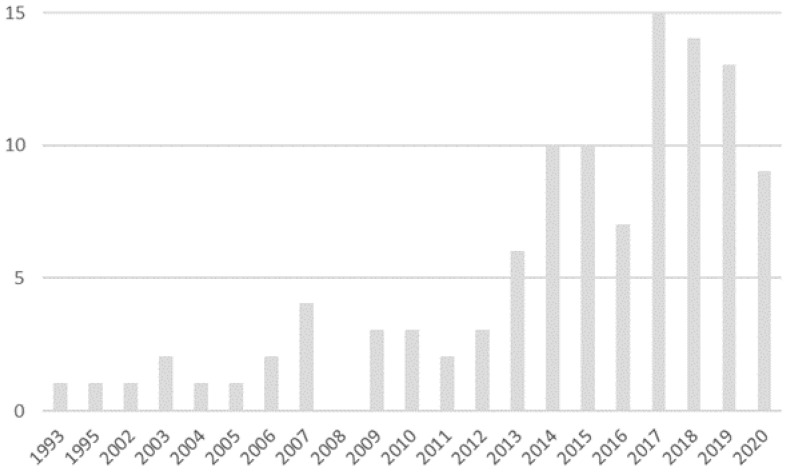
Number of articles per year.

Of the 109 articles finally selected, it is relevant to indicate that from 2014 onwards, annual references to e-health in carers of people with Alzheimer’s disease doubled with respect to previous years, with 2017 being the year with the greatest number of publications on that topic (Figure 3).

### 3.1. Caregiver Education and Support

Various studies indicated that, in particular, ICs need information on medication management and possible behavioral changes in PwD, since they are medically unqualified and incapable of coping with changes in PwD behavior [28,32,33,34]. Therefore, different programs and online devices focused on ICs have been developed.

#### 3.1.1. Surveillance Devices

- Design

The most basic interventions for informal caregiver support have been performed using remote surveillance technology. Zwierenberg et al. [35] used the Livind lifestyle-monitoring system in the homes of PwD living alone. Users were affected by different levels of dementia, from very mild to moderately severe. This surveillance system uses passive infrared (PIR) sensors in every room of the house, along with door-contact sensors placed on the front and back doors. The PIR and door sensors are connected wirelessly to a centralized database that can be accessed through the internet on a web-based dashboard.

- Results

After an intervention of, on average, 6.5 months, ICs stated that the surveillance system provided some benefits for caregiving, such as reassuring the caregiver, improving their performance as informal caregivers, and promoting a sense of safety in the PwD. However, although the surveillance system was built upon the needs that ICs reported on personal interviews, Zwierenberg et al. [35] did not provide data on how the expertise of ICs was improved with regard to facing problems related to medication or behavioral changes in PwD.

- Conclusions

Although surveillance systems can be user-friendly and might increase confidence and peace of mind, benefits are often associated with risks in a caregiving situation [36]. Thus, surveillance technologies might be a good add-on in a caregiving situation to maintain the independence of PwD living alone; however, these systems do not provide educational resources to ICs to face difficulties that might arise in caregiving situations.

#### 3.1.2. Telephone-Based Technology

- Design

The lowest level of intervention focused on ICs has been provided by telephone-based technologies. These interventions have been delivered mostly through telephone conferences with other caregivers, although some programs developed individualized interventions for individual informal caregiver. Czaja et al. [37] reported the use of a computer-integrated telephone system (CTIS) designed to augment a home-based family therapy intervention. According to Czaja et al., “The goal of the SET intervention is to identify specific family interactions that may be linked to caregivers’ burden, to empower the family by teaching them new ways to communicate with each other, and to restructure the family’s interactions”. This system involves the use of screen phones that allow both text and voice to be sent during an interactive session. With this CTIS, ICs can place a phone call to a family of friends, can participate in group sessions with trained professionals and other ICs or leave messages, and can receive reminders from therapists.

Another telephone-based system is Dementelcoach, was described by van Mierlo et al. [38]. This 20-week telephone support intervention was developed to offer tailored support through telephone calls from professional caregivers who provided emotional, social, and practical help to ICs of PwD. Professional caregivers provided support to ICs about using coping strategies in different areas of experience, such as coping with behavioral changes.

- Results

Regarding the CTIS, Czaja et al. [37] found that ICs rated the system as satisfactory and found it easy to use, although differences were found when comparing Cuban Americans with white Americans. Again, no data were reported about the efficacy of the CTIS to improve caregivers’ ability to cope with medication and behavioral changes in PwD. Regarding the Dementelcoach, van Mierlo et al. [38] reported an increase in feelings of competence and a decrease in mental health complaints in ICs after the Dementelcoach intervention. However, van Mierlo et al. did not report how the increase in feelings of competence was associated with improvements in medication management or coping with behavioral changes in PwD. This could be partly due to the fact that a small proportion of ICs receiving Dementelcoach reported to have received new information or practical support [39].

- Conclusions

Overall, neither surveillance systems nor telephone-based interventions seem useful to improve the competence of ICs in coping with medication management or behavioral changes in PwD.

#### 3.1.3. Web-Based Technology

According to the literature reviewed, the different online programs for IC skill training can be classified into two groups (Table S1): information platforms and training platforms.

- Design

Informational platforms are the most basic online platforms for ICs, which provide information about changes associated with dementia from both a cognitive and a behavioral or social perspective. These platforms employ different techniques such as informal caregiver forums [40], web portals, or computer programs [41,42,43,44,45,46,47,48]. The Talking Point is a discussion and support forum where ICs can seek help and find discussions about care-related issues, share information, or receive advice. Online training and support programs contain different modules for IC training, mainly prepared by healthcare professionals [41,43,44,45,46,47]. Generally, online training programs include modules with information about the different aspects of dementia [39,40,41,42,43,44,45], changes related to BPSD [42,47,48], the possibility of establishing social contact with professionals or other ICs [41,42,43,44,45,46], and reminders/calendars of the caregiving tasks in relation to the person with dementia [43,44,45,46,48].

Training platforms are the most complex level of interventions for ICs of PwD related to online programs or mobile applications that allow professionals and ICs to report on intervention strategies for different caregiving situations. The methodology of these interventions ranges from programs based on chat groups with videos and teaching material [49] to web-based interventions with information modules, chats, and videoconferences with professionals and other ICs [33,50,51,52,53,54,55,56,57,58,59]. One of these programs, developed by the WHO (iSupport), has been translated into several different languages to adapt it to different media and cultures [28,60].

The main characteristic of these programs is that, besides providing information about dementia and changes associated with dementia such as behavioral and psychological symptoms of dementia (BPSD) [33,49,50,51,52,54,55,58,59,61,62] and promoting SS with professionals and caregivers [33,49,50,55,56,57], they also provide information about how to cope with changes associated with dementia by means of practical strategies using videos, chats, and home-based tasks. The Alzheimer’s Caregiver Support Online [61] provides an Electronic Library with information on caregiving techniques; Dealing With Dementia [62] provides text material and videos that model positive caregiving strategies; Caring For Me [49] includes videos on managing caregiving tasks; Diapason [50] provides caregivers with skills to manage daily life difficulties; the Internet-Based Savvy Caregiver Program [51] includes two modules focused on providing practical help and on managing daily care and difficult behaviors; iSupport [52] includes a module on providing everyday care; the Mastery Over Dementia program [53] includes text material and videos along with exercises and the guidance of a coach for coping with behavioral problems; the Rhapsody [33] program includes a chapter focused on dealing with behavioral change and problem solving with regard to medical information; the SCORE program [54] includes video vignettes and written information on skill training to manage common behaviors associated with dementia; the STAR program [55] includes a module on practical difficulties in daily life and how to help by best practice; the Tele-Savvy program [56] includes videos and group videoconferences where caregivers can share and learn strategies for guiding a person through activities of daily living or for designing tasks or activities to keep the person contentedly involved during the day; the UnderstAID program [58] includes modules on daily tasks or behavioral changes; the WeCareAdvisor program [59] provides ICs with caring strategies and evaluates how the strategies work for them.

- Results

Empirical evidence about the effectiveness of informational platforms for ICs of PwD focuses on variables related to burden [41], depression [41], QoL [41,48], knowledge about dementia [42,47], feelings of competency to provide care [42,45,48], satisfaction with care [45], and SS [45,46]. The results show that online information and support for ICs of PwD increases ICs’ knowledge about dementia [41,42,44,47] and may be effective in improving ICs’ QoL [41], raising a feeling of competence in providing care [44,45] and maintaining perceived SS [45].

To assess the effectiveness of training platforms in improving the care given to the person with dementia and the status of ICs, the study variables of these programs focus on the perceived personal effectiveness of the caregivers [33,49,50,52,53,55,56,58,61,62], caregiving skills [51,53,56,62], symptoms of stress, anxiety, and depression [33,49,50,52,53,54,55,56,58,59,61,62], perceived burden [33,50,52,54,55,56,61], quality of life [33,52,55], and health [49,50]. In addition, relevant outcomes of these intervention programs include appraisal [50,52,61] and knowledge about dementia [50,51,55]. Studies based on these programs yielded contradictory results. With regard to variables relating to dementia care, some studies found an increase in knowledge about dementia [50] and in self-efficacy [49,56,61,62], caregiving skills [51], and appraisal [61] of ICs. With regard to psychological and health variables, several studies found improvements in symptoms of anxiety and depression [49,53,55,56,58,62], burden [56], and caregivers’ health [49]. Others, however, found no benefit from interventions based on online programs in increasing knowledge about dementia [55], self-efficacy [50,53,55,58], caregiving skills [53], or appraisal [50]. Likewise, they did not find any improvements in symptoms of anxiety and depression [49,50,54,59,61], burden [50,54,59,61], and caregivers’ health [50]. Overall, none of the studies reported data on ICs’ skills regarding medication management. With respect to strategies for coping with behavioral changes, positive results were reported for the Alzheimer’s Caregiver Support Online [61] and the Internet-Based Savvy Caregiver [51] programs.

Data about the effectiveness of the different programs for training ICs focus on variables related to subjective relationships concerning health, personal effectiveness for providing care, symptoms of anxiety or depression, or burden. However, the most useful information about the effectiveness of these interventions would come from analyzing both subjective and objective changes in the skills learned by the ICs of PwD. Thus, in addition to providing subjective data on perceptions about health and skills for coping with caregiving, it would be useful to analyze whether informal caregiving training programs improve the quality of caregiving through variables directly related to caregiving. These data would provide information about the lack of effectiveness of these programs in improving the care provided by ICs. Pleasant et al. [42] included an objective measure of the effects of caregiving training. In the study of the CARES platform, participants were shown a video where a caregiver helped a woman with dementia insert her dentures before a meal. Participants were asked to mark the caregiving techniques that demonstrated person-centered care out of a list of eight options (five were correct). Their results showed that, even though knowledge about dementia improved after the intervention, no significant effect was found on identifying the correct video caregiving items. Duggleby et al. [48] reported no differences in the cost of individual health and social services or in overall service costs after 3 months of intervention.

- Conclusions

Although both informational platforms and training platforms might have a positive impact on ICs of PwD by increasing knowledge about dementia, reducing depression, anxiety, and burden, and improving the quality of life of ICs, the few objective measures related to the quality of care seem to suggest that these benefits do not transfer to the quality of the care provided. Thus, the effects of both informational platforms and training platforms on the ICs’ skills to cope with medication and behavioral problems of PwD are, at best, uncertain. More research is needed that includes objective measures of medication and behavioral problems management to analyze the impact of web-based platforms on the ICs’ skills for caring.

### 3.2. Social Support for ICs in Online Platforms/Interventions

In the present work, we use the term SS as “the function of social relationshipsthe perception that social relationships will (if necessary) provide resources such as emotional support or information” [63]. Given the numerous interventions in which the need of SS for ICs of PwD is addressed as a necessary and specific dimension, we undertook an analysis of the online interventions that include SS for ICs among their components as one of the differentiated variables. Of the 109 articles analyzed, 39 studies provided data about SS for ICs. We did not include platforms that reported SS as one component of the intervention if data on SS were not reported. Four intervention modalities were established based on the criterion “modality or type of intervention”, and a fifth modality in which studies were grouped based on the online “REACH” program.

#### 3.2.1. Interaction with Other ICs and/or Health Professionals

- Design

In these platforms, a multicomponent intervention is provided (Table S2), by which ICs are trained in the management of PwD and that also include a module aimed at improving ICs’ psychoemotional wellbeing. In the case of the “Inlife” platform [45], for example, the whole program is devoted to “care for the caregiver”. On these platforms, ICs interact with each other and with healthcare professionals who act as moderators. We included within this category both web platforms and the program by Poole et al. [47], which is an MOOC (Massive Online Open Courses) with similar characteristics.

- Results

Users reported that they felt comfortable in these “safe” spaces as they allowed them to interact with people going through similar experiences, perceiving the support of their peers. The participation in, or moderation of, the interactions by a healthcare professional was reported as a positive aspect, allowing ICs to interact freely in the knowledge that inappropriate comments would not take place [43,47,64,65]. The results of the interventions showed improvements in perceived SS and/or reduced feelings of loneliness [45,64,66,67], improved quality of life [41,65], and reduced stress [49,66,68]. Despite these results, the researchers showed that, on occasions, improvements reported by the ICs in appraisals of a qualitative type [67,69,70] were not reflected by quantitative analysis results. One aspect worth noting is that a different use of the platforms was observed according to the age of the ICs, with younger ICs making greater use of them [71] and, therefore, obtaining greater benefit from having more extensive online social networks [45].

- Conclusions

As a recommendation for improvement, it is suggested that existing social networks, such as WhatsApp, Facebook, Instagram, and Twitter [45,58,67], be used in the interactive modules between ICs since, as indicated by Boessen et al. [43], different care organizations and companies develop their own platforms, and it is complicated for ICs to learn how to use these new tools.

#### 3.2.2. Training without Interaction

- Design

With this type of program (Table S3), which does not establish a schedule for training, ICs can freely access the websites when their care tasks or other obligations allow them to do so.

- Results

The results obtained are not very encouraging, since most of the studies reported no changes or improvements in variables related to SS between the experimental and control groups [48] or in the intention to obtain SS in the pre–post measures [62].

- Conclusions

One of the pitfalls of these programs is the high rate of dropouts, due to the caregiving tasks themselves or for technical reasons [52,72].

#### 3.2.3. Online Support Groups

- Design

The programs included in this category (Table S4) encompass those group interventions in which ICs can interact with the other members of the group. Furthermore, ICs can interact with a healthcare professional who intervenes to moderate the meetings in a more or less supervisory way according to the objectives of the program. One exception is the program reported by Lindauer et al. [73], in which the intervention for the IC is provided by a therapist on an individual basis. The success of group interventions lies in the fact that the participants interact with people going through circumstances that are similar to their own [74]. This success is also evident when the interventions are online, since, from the studies analyzed, the results indicated a reduction in loneliness and an increase in SS for ICs [37,47,49,50,51]. Online support groups can be delivered via videoconference [73,75,76] or by telephone [77,78,79,80].

- Results

Comparing videoconference and telephone online support groups, it seems that better results are obtained via videoconference, since no differences were observed between the intervention and the control groups in the telephone interventions [77,79]. The only significant finding in the telephone interventions is the one reported by Winter and Gitlin [79], who found a reduction in depressive symptomatology in a group of older women. Winter and Gitlin indicated that this could be due to the fact that this group of people was the most isolated. McHugh et al. [78] pointed out that, although the provision of SS through telecommunications is ideal for some ICs, visual cues are essential for the optimal development of social relationships. In the study by Marziali and Garcia [49], when two intervention methods (based on a support group either via chat or via video) were compared, improvements in the mental health of the group involved in the video intervention were observed. Marziali and Garcia pointed out that the participants could think on the significance of the emotional support received and benefited from the advantages offered by taking part in a self-help group.

- Conclusions

One suggestion made by users of online support groups, via both telephone and videoconference, is the possibility of maintaining social interaction between members for a longer period of time after the intervention [76,78]. Likewise, several authors suggested that, in order to maximize the effectiveness of these interventions, these programs must be specialized and tailored [77], and the composition of the groups should be very similar as regards the characteristics of both the ICs and the PwD [76]. Suggested methodological improvements include the need to increase the size of the sample in order to generalize the positive results observed [76].

#### 3.2.4. Online Forum

- Design

This modality includes research analyzing the interactions of ICs in online forums specialized in dementia (Table S5). Currently, 86% of ICs use internet [6] and, therefore, already have access to these online peer-to-peer communities. Users report that these platforms are used as a source of SS [81] as they allow them to share experiences with people who are living similar situations and, thus, feel support from the group [6,40]. As with other types of peer support programs, sharing experiences “are a good form of support to help reduce the negative impact of a disease” [82]. Another modalities include the research conducted by Bateman et al. [23] in which two methods of information exchange (crowdsourcing or friendsourcing) through social networks (Facebook) between ICs, and the role of peer-patrons in an online peer support group for ICs of people with Alzheimer’s disease [6].

- Results

A trend towards an increase in emotional and informational support related to the use of online forums and peer patrons peer support groups was reported. Peer-patrons may be a source aiding ICs in adapting and dealing with their changing circumstances, since peer-patrons have a unique way of interacting with other ICs, and their contributions can play an important role in making online healthcare peer support portals [6].

- Conclusions

In order for online forums interventions to be implemented on social networks, “participants’ responses indicated a need for additional support, as well as a familiarity with Facebook that might make it a more appealing source of support than anonymous forums or crowdsourcing” [23]. Limitations of these online programs were the limited use of the platform [40] and difficulties in generalizing the results among ICs that used blog versus those who did not. Some ICs do not have regular access to the Internet [81], and that is why “one of the key needs identified to inform future design of an Alzheimer’s Caregiver Forum was a design sensitive to the capabilities of its elder user audience” [83].

#### 3.2.5. The REACH Program

- Design

Resources for Enhancing Alzheimer’s Caregiver Health (REACH) was created in 1995 as a research program on interventions offered to improve the care situation of people diagnosed with Alzheimer’s or other dementias. The program combines multiple treatments with the purpose of assessing their effectiveness individually and as a whole.

It was first used in the USA (Boston, Birmingham, Memphis, Miami, Palo Alto, and Philadelphia) and has since been used in many countries such as Korea, Hong Kong, and Germany, in aging programs and by associations that focus on PwD or similar (Table S6). Although the REACH program is based on various theoretical frameworks, these are all included within the health-stress model, its main objective being to alleviate the stressful effects that affect the health of ICs. Specifically, the system is intended to facilitate linkages between ICs and other family members, friends, and other ICs as well as to facilitate access to information on available resources [37]. It is a multicomponent intervention that includes, among its treatment areas, individualized information and support strategies for caregivers; group support and family therapy; psychoeducation and specialized training for caregivers; technological support systems and tools focused on the home.

- Results

Several interventions were based on some of the components of the original REACH program, in pursuit of a faster application or the specific treatment of one of the target areas, such as SS. The results in this area showed that ICs increased their SS and reduced loneliness [76,84,85,86], although these changes were more significant in ICs that had more extensive support networks before starting the intervention [84].

- Conclusions

These studies show that interventions using ICT are useful for reducing social isolation in ICs, which is even more accentuated in rural areas or areas with mobility limitations. These results are consistent with those of other interventions conducted face to face [86]. Therefore, these strategies could be a useful alternative for eliminating the time and space barriers encountered by ICs when it comes to attending these intervention groups.

## 4. Discussion

The present review was developed with the aim of analyzing the efficacy of e-health programs for ICs. The results show that e-health programs increase ICs’ knowledge about dementia and perceived SS. Through e-learning platforms, including videos, texts and vignettes [33,42,43,44,47,48,49,50,52,53,54,55,56,58,59,62], ICs can learn new skills for the caring of PwD, whereas the use of web platforms, online support groups and online forums improve the SS for ICs by increasing the interpersonal contact with both ICs and health professionals. As a consequence, e-health programs might have beneficial mental health effects on ICs of PwD. However, the increase in knowledge about dementia and the improvements in mental health and quality of life do not seem to have an impact on the quality of the care provided, as no evidence of improvements in objective outcomes was found. In the time of SARS-CoV-2, as obvious, the use of the Internet and these programs has accelerated, and these interventions will continue to be an efficient and convenient alternative to live training [39]. Given that caring for PwD is a complex endeavor, the cumulative stress of providing care during a global pandemic further beckons the need for training to reduce ICs’ burden and to improve mental health, with the ultimate goal of providing better care for PwD.

Regarding the effectiveness of online interventions in providing SS to ICs, our results show that it is important that group members share similar characteristics, as regards to both ICs and those being cared of, as this increases group cohesion [54]. Likewise, for there to be a climate of safety and confidentiality, the participation of a group moderator is key (whether a health professional or a peer-patron), as they help to increase cohesion and reduce the risk of demotivation and the lack of adherence to the group intervention. Lastly, with regard to the objective of online platforms focused on SS, it is suggested that these platforms include social networks that users are already familiar with (e.g., Facebook, WhatsApp) [43,67]. Thus, ICs do not need to learn how to operate new platforms but employ those they already use with their friends and family networks.

One of the most relevant findings of this review is the limited number of objective measures used to assess SS. It is, therefore, recommended that both a module for intervention in this dimension and a specific assessment be included in e-health programs. This assessment should be carried out both qualitatively and quantitatively, systematically using instruments that have already been designed and whose validity have been proven for analyzing SS, such as The MOS SS survey [87], the PSSC Perceived SS Caregiving [88], the MSPSS Multidimensional Scale of Perceived SS [89], and the Lubben Social Network Scale [90]. Thus, effectiveness could be compared between interventions developed via videoconference or avatar [91], which have been shown to be more satisfactory than those performed by telephone [49,78].

One of the main limitations on identifying the effectiveness of the interventions of the studies reviewed is the difference in the duration of the interventions. While some programs lasted for 30 days [62], others lasted for 16 weeks [45,55,61], 6 months [41,49,53,54], or even up to 18 months [76]. In the context of neurodegenerative diseases, it can be difficult to find a balance between the duration of the intervention and the effects on the care provided by ICs. While short interventions may not provide an appropriate learning context for improving care, long interventions may not show significant effects due to the degenerative nature of the diseases and patient’s progressive decline, and so any possible beneficial effects may be nullified in long-term assessments. Notwithstanding, regardless of the duration of the interventions, future studies must include long-term follow-up assessments after the intervention has ended. Furthermore, as demanded by the users [76], ICs should be offered the possibility of taking part in support groups for longer after the intervention.

Despite the benefits provided by online support interventions for ICs of PwD, the use of these programs is more prevalent among younger ICs who have more extensive social networks [45,46,71]. This suggests that the results may not be generalizable to the whole population of ICs, since these are usually older people over the age of 60 [20,21,22].

## 5. Conclusions

The use of ICTs can be beneficial for ICs both emotionally and instrumentally by incorporating new skills to be used in the care of PWD. The main conclusions of our review are:

Given that many ICs have not yet incorporated ICT skills in their everyday activities [92], education in the digitalization to ICs of PwD is a priority objective to improve the use of online tools that help in the improvement of care and the quality of life of PwD and their caregivers.

Although surveillance devices, telephone-based technologies and web-based platforms have shown positive results for the perceived psychological well-being of ICs of PwD and their feelings of mastery for caregiving, e-learning tools have not yet shown efficacy through objective outcomes for improving the quality of the care provided by ICs. Objective measures of effectiveness should be included when assessing the efficacy of eHealth interventions for ICs of PwD.

Given the limited time available to ICs and the low mastery of NNTT, platforms specifically developed to improve the SS of ICs would benefit from incorporating tools that ICs are familiar with (such as Facebook, or WhatsApp).

In SS improvement programs for ICs, offering the possibility that ICs can interact with health professionals or moderators is essential to make the users feel comfortable in this “safe” space [43,47,64,65,73,78], and, therefore, to improve the perceived SS when using these e-health interventions.

A harmonization of intervention protocols is encouraged in order to reduce the heterogeneity in duration of interventions, outcomes, or follow-up assessments.

## Data Availability

Data are available upon request.

## Supplementary Materials

The following are available online at https://www.mdpi.com/article/10.3390/ijerph18157728/s1. Table S1: Online programs for informal caregivers: training and pharmacological treatment, Table S2: Webs platforms with interaction with other IC and/or health professionals, Table S3: Webs platforms with training without intervention, Table S4: Online support groups, Table S5: Online forum, Table S6: REACH. (Resources for Enhancing Alzheimer’s Caregiver Health Project).
