# Supplementary material for: Effects of e-Health Training and Social Support Interventions for Informal Caregivers of People with Dementia—A Narrative Review
Source: Int J Environ Res Public Health. 2021 Jul 21;18(15):7728. doi: 10.3390/ijerph18157728 (PMC8345641; doi:10.3390/ijerph18157728)
Supplement: Supplementary file 1 [file ijerph-18-07728-s001.zip › ijerph-1222615-supplementary.pdf]

International Journal of
Environmental Research and Public Health

MDPI

Table S1. Online programs for informal caregivers: training and pharmacological treatment

Authors	Program	Objectives	Material	Persons involved	Timing	Primary outcomes	Secondary outcomes	Results
Torkamani et al. (2014)	ALADDIN	To offer avenues of support and information to the carer. It also facilitates distant monitoring	Four key features: 'ALADDIN TV', 'SOCIAL NETWORKING', 'MY TASKS', and 'CONTACT US'.	IC;HP	3-6 months	Burden, depression, QoL,	Ease of use of the system, the user's willingness to continue using it, and recommending it to a friend (carer) or substituting it with in-person consultations (clinician)	No significant main effects or interactions on the carers' distress or depression. A significant Group x Time of Assessment interaction on QoL. Carers felt that ALADDIN gave them more confidence (64%); made them more aware of their relative's health (53%); and provided them with information about dementia (56%); reduced their anxiety about their relative's health (46%); and had the potential to improve their QoL (60%).
Gluekauf et al. (2004)	Alzheimer's Caregiver Support Online	To assess the impact of the program on the psychosocial functioning of caregiver participants, particularly their perceptions of self-efficacy, emotional growth, and burden from the caregiving experience	Six 45-minute live, interactive classes: Class 1: Basics of Dementia: Memory, Emotions, and Behavior<br>Class 2: Relaxation Training<br>Class 3: ABCs of Positive Caregiver Thinking<br>Class 4: Increasing Caregiver Emotional Well Being<br>Class 5: Assertive Responding in Caregiving Situations	IC;HP	16 weeks	Self-Efficacy, Stress, Appraisal (caregivers' perceptions of the demands and rewards of their role and role-related activities)		Substantial improvements were noted in the perceptions of self-efficacy in performing routine caregiving duties and managing challenging care recipient behaviors, as well as the appraisals of the emotional caregiver burden from the pre- to the post-testing phase. In contrast,

			Class 6: Managing Challenging Caregiving Situations					caregivers reported little or no change on the positive dimensions of the caregiving experience (i.e., stress-related growth and positive caregiving appraisals), or their perceptions of time burden in providing caregiving assistance
Beauchamp et al. (2005)	Caregiver's Friend: Dealing with Dementia 30	to create a multimedia Internet intervention to offer ongoing worksite support to employed family caregivers of persons with dementia	Caregiver's Friend: Dealing With Dementia is a Web-based multimedia intervention that provides text material and videos that model positive caregiving strategies. Program content was created for this project by research scientists and instructional designers on the basis of an extensive literature review; interviews with academic gerontologists, social workers, nurses; and focus groups with an ethnically and geographically diverse range of family caregivers. The components of knowledge, cognitive, and behavioral skills, and affective learning are presented across three modules developed from formative data: Being a Caregiver, Coping With Emotions, and Common Difficulties. In the	Professionals	30 days	The outcomes of interest were caregiver strain, caregiver gain, depression, and anxiety.	Self-Efficacy (confidence to do a good job caring for their care recipient; to juggle their many roles; to deal with the stress of caregiving; to make a plan to address a problem with their care recipient; to resolve any problems with their care recipient; and to turn to their family for help). Coping Skills (problem-focused strategies and social support). Satisfaction Survey	Compared with the control group, treatment participants reported significantly greater gains with respect to the measures of self-efficacy, intention to get support, and caregiver gain. In addition, the treatment participants reported significantly greater reductions in caregiver stress, caregiver strain, depressive symptomatology, and state anxiety compared with the control participants. The only scale that did not differ with respect to pretest–posttest change across conditions was the Ways of Coping scale, which measured self-reported frequency of

			Common Difficulties module viewer coping with the later phases is presented with strategies and dramatizations on handling difficult behaviors such as aggression, paranoia, anxiety, and flat affect.				employing specific stress-reduction strategies
Pleasant et al. (2017)	CARES	To evaluate the CARES® program training among formal and informal caregivers	CARES® of four modules: (1) Meet Clara Jones, an introduction to person centered care; (2) introduction to dementia; (3), understanding behavior as communication; and (4) the CARES® approach to Connect, Assess, Respond, Evaluate, and Share with other team members when providing care.	HP	Not reported	Dementia knowledge, competency,	Performance on the dementia knowledge questionnaire improved from pre-test to post-test and from pre-test to follow-up. No significant effect was found among the video caregiving items, either on correctly endorsed, or incorrectly endorsed items.
Marziali & Garcia (2011)	Caring for me	To examine dementia caregivers' responses to using technology	"(1) a text-based Chat Group intervention (Chat Group) that included access to a caregiver information handbook and 6 videos on managing caregiving tasks; (2) an online video	IC;HP	6 months	caregiver health, depressive symptoms, caregiving distress	Video Group showed (a) significantly greater improvement in mental health, and (b) lower distress scores associated with managing the care recipient's deterioration in mental (cognitive) function. In contrast, the Chat Group had lower distress scores associated with managing IADL.
Boessen (2017)	Cubes platform	To test usability and perceived value of this platform by	The integrated platform consisted of standard Cubes (e.g., "Contacts/Clients," "Messaging," and "Calendar"), Cubes	IC;HP	10 weeks	Post-Study System Usability Questionnaire (PSSUQ) and	Daily use of the platform reported by most of the caregivers. Family caregivers more easily and

		family and professional caregivers of PWD	for care related information (e.g., "Info Dementia," a care record called "Care Notebook," "ShareCare," and "Medication plan/reminder"), and, only for family caregivers, Cubes for entertainment ("Radio," "YouTube," and "My Games"). After four weeks, a "Videocall" service was added to the platform of both family and professional caregivers			semi-structured interviews		quickly informed about the situation of the PWD. Family caregivers felt calmer and more prepared before or without visiting the PWD.
Cristancho-Lacroix et al. (2015)	Diapason	To evaluate the impact of the Diapason program on caregivers' perceived stress	Delivered in a free, password-protected, fully automated website. The intervention targeted (1) caregivers' beliefs about the illness and the caregiving role, (2) caregivers' skills to manage daily life difficulties, and (3) caregivers' social support and help-seeking behavior to obtain respite or financial support, and to meet and discuss with peers through a forum. Twelve thematic sessions were sequentially and weekly unblocked once the previous one was entirely viewed	HP	12 weeks	perceived stress of caregivers	Self-efficacy, Perception and reaction to cognitive or behavioral symptoms of PWAD, Subjective burden, Depressive symptoms, Self-perceived health	Tests did not show significant differences between the experimental and control groups over time. Only the VAS evaluating knowledge of the disease showed significant change at month 3 but not at 6 months
Hattink et al. (2016)	Digital Alzheimer Center	To offer comprehensive information on dementia, to enhance social activities, support peer-to-peer	The DAC offers a comprehensive menu containing information on diseases, an overview of appointments and dossiers, community sections,	IC;HP	Not reported	The type of errors participants made before reaching the end-goal, The		The content of the DAC was rated understandable and clearly written by carers (82%). 78% carers indicated that the DAC

		contact, and to provide easy access to communicate with health care professionals.	and information on upcoming events and news. In a specially secured section, patients can email their health care professionals at the Alzheimer Center and view their appointments and medical correspondence			number of errors; and Time on task	was "very useful" or "useful" and that it had an added value over the regular care offered by the center. Participants indicated that the DAC was especially useful for understanding dementia and for dealing with dementia
Lai et al. (2012)	Ginkgo	Not reported	Not reported	Not reported	7 weeks	Depression, General Health, Alzheimer's Disease Knowledge Burden, Quality of Life	Knowledge gained by the online workshop participants after attending the workshop was significantly greater. No significant difference was found in the online group pre-intervention and post-intervention. The online group participants mentioned that what they gained the most from the online workshop was knowledge and skills.
Dam et al. (2017)	Inlife	The development and piloting of the Inlife intervention	Inlife aims to lower the threshold to seek support, prevent feelings of loneliness, improve social support, caregiver competence, and access to information. The content is focused on developing positive social interactions and promoting the involvement of the personal care networks in daily life and care activities.	IC;HP	16 weeks	Perceived support, received social support, feelings of loneliness, feelings of being capable of caring on three domains: (1) satisfaction with the individual with dementia as a recipient of care, (2) satisfaction with one's own	The data indicated a trend towards improvements in feelings of competence and a decrease in feelings of loneliness (in the high-active Inlife users)

			Participants can post comments on the website			performance as a caregiver, and (3) consequences of involvement in care for the personal life of the caregiver.		
Dam et al. (2019)	Inlife	To evaluate the internal and external validity of Inlife intervention	The program participation questionnaire (PPQ) and semi-structured interviews	IC;HP	16 weeks	Feasibility of and satisfaction with the Inlife platform		People were sometimes inclined to use WhatsApp instead of Inlife because it was more routinely used in their daily practice. Caregivers reported that guidance by volunteers or coaches in the community would have been valuable to provide additional user guidance and awareness of social support opportunities
Lewis et al. (2010)	Internet-Based Savvy Caregiver	To provide persons caring for people with dementia the knowledge, skills, and outlook they need to undertake and succeed in their caregiving role	The IBSCP consists of 4 modules: (1) the effects of dementia on thinking; (2) taking charge and letting go; (3) providing practical help; and (4) managing daily care and difficult behavior.	IC;HP	5-6 weeks	Effect of the program on caregiver skills, strategies, and knowledge		45% responded with learning strategies or techniques for dealing with the behaviors associated with dementia, followed by 12% identifying more knowledge and understanding the behaviors they are experiencing
Mehta et al. (2018)	iSupport		To establish the effectiveness of an online self-help training and support program (iSupport) compared to an education only comparison condition (EOC) which provides	IC;HP	3 months	Burden and symptoms of depression and anxiety	Caregiver quality of life, person-centered attitude, self-efficacy and mastery	Intervention protocol. No results provided

			education about dementia and caregiving through an on-line e-book					
Pot et al. (2019)	iSupport	The content of iSupport is based on the Kitwood's model, in which the personhood of someone with dementia is central, and in which care is essentially thought of as interaction, according to each individual's needs, personality and ability	The online programme includes five themes: a) what is dementia (one lesson); b) being a caregiver (four lessons); c) caring for me (three lessons); d) providing everyday care (five lessons); and e) dealing with changing behaviour (ten lessons).	IC;HP	Not reported	Not reported		Descriptive article
Teles et al. (2020)	iSupport	The development of an adapted version of iSupport to the Portuguese culture	The adaptation process included five major steps (cf. Figure 2): (1) needs assessment; (2) content translation and technical accuracy check; (3) cultural adaptation; (4) expert panel appraisal; and (5) fidelity check	IC;HP	3 months	Burden	"(i) symptoms of depression and anxiety, (ii) quality of life, (iii) positive role appraisals mediating between the stressor	Intervention protocol. No data reported
Xiao et al. (2020)	iSupport	To identify stakeholders' perspectives on adapting the WHO iSupport programme for use by informal caregivers	A semi-structured focus group discussion guide based on the WHO 'iSupport version 1.0. Adaptation and Implementation Guide' was developed. Informal caregivers and care staff participated in separate focus groups	IC;HP	Not reported	Final themes for the program		Informal caregivers perceived iSupport as an opportunity to provide an online one-stop shop to meet their education needs and their needs to manage care services.

		of people with dementia in Australia						
Poole et al. (2020)	Massive Open Online Courses	To provide sensitive information about aspects of dementia progression identified as confusing, misunderstanding and distressing for families	Through a range of media including videos, articles, quizzes, images and discussion forums; family carers, health and social care professionals, and the research team shared key information with learners.	IC;HP	Not reported	Not reported		Over 90% of learners reported acquiring new skills and knowledge, and over two-thirds have applied this learning in their daily lives. Over three-quarters also report sharing what they have learned with others.
Blom et al. (2015)	Mastery over Dementia	To reduce caregivers' symptoms of depression and anxiety	8 lessons and a booster session with the guidance of a coach. Each lesson consists of information (text material and videos), exercises, and homework, with an evaluation at the start and end of each session	HP	5-6 months	Depression	Anxiety, perceptions of distress, memory and behavior problems, the caregivers' sense of competence, the caregivers' sense of mastery	The treatment variable had a significant effect on the outcome scores for symptoms of depression and anxiety. The differences in effect sizes between both groups were moderate for the HADS-A scores and small for the CES-D scores. No data on the other four variables
Duggleby et al. (2018)	My Tools 4 Care	To support carers during their transition experiences	MT4C consists of six main sections:<br>(1) about me, (2) common changes to expect, (3) frequently asked questions, (4) resources, (5) important health information, and (6) calendar	HP	3 months	HRQOL consisting of 12 questions, measuring 8 domains of functioning and well-being (physical functioning, role functioning, bodily pain, general health, vitality, social functioning, emotional health, and mental health)	SF-12v2 PCS (physical component summary score), General Self-Efficacy Scale, Herth Hope Index ((1) temporality and future, (2) positive readiness and expectancy, and (3) interconnectedness)	No significant group differences were observed in the primary or secondary outcome measures.

Kurz et al. (2016)	RHAPSODY	To develop a comprehensive, multi-media, educational, and skill-building program for informal carers of people with young onset (i.e. symptoms occurring before the age of 65 years) dementia	Seven chapters addressing the major components of support for carers, including medical information (with a focus on problem solving), dealing with behavioral change, coping with role shift in the family, and finding help.	IC;HP	12 weeks	Caregiving self-efficacy	Caregiver stress, Caregiver burden, problems dealing with behavioral change, Health-related quality of life.	Design of a pilot study-No data reported
Hicken et al. (2016)	SCORE	To examine whether an electronic-based intervention could be deployed in support of rural caregivers of veterans with dementia	The intervention material consisted of: (1) video vignettes, once per week, portraying dementia progression and caregiving skills; (2) written information about health topics and caregiving skills 2-3 times per week; and (3) brief assessments of caregiver health and well-being 2-3 times per week.	HP	4-6 months	Burden, grief, depression, family conflict and family hardship, nursing home placement		In general, there were no differences in change scores between cohorts within either participant group
Hattink et al. (2015)	STAR	To improve the knowledge about dementia for informal caregivers, volunteers, and professionals in dementia care by developing and evaluating an online training program in	8 modules on different topics in dementia care: 2 at a basic level and 6 at an intermediate and advanced level	IC;HP	2 to 4 months	Knowledge on dementia and attitudes regarding dementia. Usefulness and user friendliness.	Empathy, quality of life, burden, and sense of competence.	No statistically significant differences in the experimental or control group among laypeople and professionals on knowledge about and attitudes toward dementia. For the secondary outcome measures, statistically significant differences on several subscales of empathy: the

		different languages and at different difficulty levels.						distress subscale, empathy subscale, and perspective subscale. Significant negative effect on sense of competence of informal caregivers, which declined in the experimental group compared to the control group, implying that participants in the course felt less competent to fulfill their care task after following the course
McKechnie et al. (2014)	Talking point	To test whether users' anxiety and depression would decrease with of forum usage.	Talking point forum, and personal interview	IC	12 weeks	Depression, anxiety, relationship with the person that they are caring for, giving equal weight to positive and negative aspects		Overall, usage was low. Both the pre and post means are in the good range
Griffiths et al. (2016)	Tele-Savvy	To test fidelity and preliminary efficacy of Tele-Savvy	Six modules (on self-care practices such as therapeutic breathing and guided meditations) draw upon evidence-based practice that were taught by experienced practitioners	IC;HP	6 weeks	Caregiver burden, depressive symptomatology, state anxiety, caregiving competence, behavioral and psychological symptoms of dementia and caregivers' responses		Significant decreases in burden, depression, anxiety, and both BPSD frequency and upset and a significant increase in caregiver competence were observed
Kovaleva et al. (2018)	Tele-Savvy	To establish Tele-Savvy's efficacy in mitigating the affective impact on	3 racial/ethnic groups: African Americans, whites, and Latinos/Hispanics.	IC;HP	6 weeks	Stress, Depressive symptoms, Anxiety, Burden, Dyadic relationship, Mastery, Skill, Reward, Self-		Feasability study. No data reported

		dementia caregivers, in promoting PLWDs' quality of life, in enhancing caregiver mastery and test the mediating effect of mastery and enactment on Aim 1 & 2 outcomes				care, Health, BPSD, Mood		
Núñez-Naveira et al. (2016)	UnderstAID	To provide caregivers of people suffering from dementia, with information, skills training, a forum to develop their social network, and daily reminders.	The understAID consists on a Learning section with a database of contents organized in 5 modules with information about 15 different topics: care of a person with dementia and caring for oneself as a caregiver. Module 1, Cognitive Declines (Topics: Attention,Memory, and Orientation);Module 2, Daily Tasks (Topics: Bathing, Incontinence, Massage and Touch, and Physical Exercises); Module 3, Behavioural Changes (Topics: Anxiety and Agitated Behaviour, Depressive Mood, Manic Symptoms, and Emotional Control and Recognition); Module 4, SocialActivities (Communication andApathy and Loss of Motivation); and Module 5, You as a Caregiver	HP	3 months	Depressive symptoms, sense of competence, caregiving satisfaction with the care provided		66.7% of the caregivers assessed the understAID application as unacceptable. In the experimental group, there were a statistically significant decrease in the observed depressive symptomatology. No statistically significant differences in any of the other variables

			(Topics: Coping with Own Stress and motivation)					
Kales et al. (2018)	WeCareAdvisor	To evaluate the program one-month effect on caregiver distress and caregiver confidence	WeCareAdvisor contains three main components: a WeCareAdvisor "prescription" where Caregivers are instructed to try the strategies for one week and then evaluate how the strategies work for them; the Caregiver Survival Guide, which is a compendium of information for dementia caregivers; daily messaging	Not reported	1 month	Caregiver distress,	Caregiver stress, depression, negative communication and relationship closeness	The experimental group had a higher percentage of caregivers with improvement in distress. No differences in caregiver stress, depression, burden, negative communication, and relationship closeness

IC= Informal Caregivers; HP= Health Professionals; PC = Professional Caregivers; PwD= Persons with Dementia

Table S2. Webs platforms with interaction with other IC and/or health professionals

Authors	Name	Social support Measures	Sample	Length
Poole et al (2020)	(MOOC) Dementia care: living well as dementia progresses	Qualitative assesment	4000 persons	-
Dam et al (2017)	InLife	Qualitative assesment	23 IC	16 weeks
Dam et al (2019)	InLife	Qualitative assesment	36 IC	16 weeks
Boessen et al (2017)	Cubes for family caregivers	Qualitative assesment	7 IC<br>32 PC	10 weeks
Xiao et al (2020)	iSupport	Qualitative assesment	16 IC<br>20 HP	-
Ducharme et al (2011)	Online Stress Management Training Program	SAM *Stress Appraisal Measure* (Peacock & Wong, 1990)*	17 IC	7 weeks
Pagán-Ortiz et al (2014)	Cuídate cuidador	Luben Social Network Scale (Lubben et al, 1988)	26 IC	7 weeks
Núñez-Naveira et al. (2016)	understAID application	Qualitative assesment	61 IC	3 weeks
Torkamani et al. (2014)	ALADDIN	QOLS *Quality of Life Scale* (Flannagan, 1982)*	30 PwD<br>30 IC	6 months
Tonsaker et al (2017)	healthexperiences.ca	Qualitative assesment	16 IC	-
Boots et al (2018)	Partner in Balance	ICECAP-*O Preferences of Older People CAPability measure for Older people* (Grewal et al, 2006)*	81 IC	8 semanas
Marziali y Donahue (2006)	Caring for Others	MSPSS *Multidimensional Scale of Perceived Social Support (Zimet et al. 1988)*	66 IC	6 months

Lorig et al (2012)	Building Better Caregiver (BBC)	Qualitative assesment	60 IC	3 months
Brennan et al (1995)	ComputerLink	IESS The instrumental and exppresive social scale. (Ensel and Woelfel, 1986)	102 IC	1 year
Chiu et al (2009)	Internet-based Caregiver Support Service (ICSS)	• MSPSS Multidimensional Scale of Perceived Social Support (Zimet et al 1988)<br>Qualitative assesment	28 IC<br>10 IC	6 months

IC= Informal Caregivers; HP= Health Professionals; PC = Professional Caregivers; PwD= Persons with Dementia
**= A dimensión of Social Support of this questionnaire*

Table S3. Webs platforms with training without intervention

Authors	Name	Social support Measures	Sample	Length
Beauchamp et al (2005)	Caregiver's Friend: Dealing With Dementia	Revised *Ways of Coping Checklist* (Vitaliano et al, 1985)*	299 IC	30 days
Duggleby et al (2018a)	My Tools 4 Care	HRQOL *Short-Form 12-item health survey version 2 (Ware et al, 1996)** Qualitative assesment	154 IC	3 months
Duggleby et al. (2019)	My Tools 4 Care	HRQOL *Short-Form 12-item health survey version 2 (Ware et al, 1996)** Qualitative assesment	72 IC	3 months
Pot et al (2015)	Mastery over Dementia (MoD)		66 IC	6 months
Duggleby et al (2018b)	My Tools 4 Care	HRQOL *Short-Form 12-item health survey version 2 (Ware et al, 1996)** Qualitative assesment	37 IC	2 months

*IC= Informal Caregivers; *= A dimensión of Social Support off this questionnaire*

Table S4. Online support groups

Authors	Name	Social support Measures	Sample	Length
Winter et al (2006)	TELESUPPORT GROUP	6-item scale adapted from Kaye's Gain Through Group Involvement Scale	103 IC	6 months
Marziali y Garcia (2011)	Caring for me	MSPSS *Multidimensional* Scale *of Perceived Social Support (Zimet et al. 1988)*	91 IC	20 weeks
Lindauer et al (2018)	STAR-C Telemedicine	Qualitative assesment	14 IC	8 weeks
Martindale-Adams et al (2013)	CONNECT – Telephone Support for Dementia Caregivers	19 social support items measured received support and negative interactions (Krause, 1995), Satisfacción (Krause, 1995; Krause & Markides, 1990), and social networks (Lubben, 1988).	154 IC	1 year
Guerriero et al (2014)	-	QoL SF-36 Short-Form *(Stewart, Hays, & Ware, 1988)**	5 IC	6 months
Berwig (2017)	Talking Time *(Protocol)*	PSSC *Perceived Social Support Caregiving* (Goodman, 1991)<br>SCS *Social Conflict Scale (*Goodman, 1991)<br>Qualitative assesment	-	6 months
McHugh et al (2012)		Qualitative assesment	8 IC	6 weeks
O'Connell et al (2013)	Telehealth Saskatchewan	Qualitative assesment	10 IC	18 months

*IC= Informal Caregivers; *= A dimensión of Social Support off this questionnaire*

Table S5. Online forum

Authors	Name	Social support Measures	Sample	Length
Narasimha et al (2019)	Peer Patrons	Qualitative assesment	1405 posts	2 months
McKechnie et al (2014)	Talking Point	Qualitative assesment	61 participants	12 weeks
Anderson et al. (2017)		Qualitative assesment	2345 posts analysed in 9 forums	2 years
Scharett et al (2017)	ALZConnected.org	Qualitative assesment	250 posts and related responses	1 year
Bateman et al (2017)	Comparing Crowdsourcing and Friendsourcing	The MOS *Social Support survey.* (Sherbourne & Stewart, 1991)	4 IC	6 weeks

IC= Informal Caregivers

Table S6. REACH. (Resources for Enhancing Alzheimer's Caregiver Health Project)

Authors	Social support Measures	Sample	Length
Czaja et al (2002)	Ad-hoc usability questionnaire	44 IC	6 months
Finkel et al (2007)	3 items from the Inventory of Socially Supportive Behaviors (Barrera et al, 1981)	46 IC	6 months
O'Connor et al (2015)	Qualitative assesment	7 IC	8 weeks
Czaja et al (2013)	10 ítems from Social Support Scales (Barrera et al, 1981; Krause, 1995; Krause y Markides, 1990).	110 IC	5 months
Bank et al (2014)	Ad-hoc scale	41 IC	12 months

IC= Informal Caregivers
